# Antithrombotic Potential of Tormentil Extract in Animal Models

**DOI:** 10.3389/fphar.2017.00534

**Published:** 2017-08-15

**Authors:** Natalia Marcinczyk, Dominika Jarmoc, Agnieszka Leszczynska, Agnieszka Zakrzeska, Karol Kramkowski, Jakub Strawa, Anna Gromotowicz-Poplawska, Ewa Chabielska, Michal Tomczyk

**Affiliations:** ^1^Department of Biopharmacy, Medical University of Bialystok Bialystok, Poland; ^2^Department of Biotechnology, School of Medical Science Bialystok, Poland; ^3^Department of Pharmacognosy, Medical University of Bialystok Bialystok, Poland

**Keywords:** *Potentilla erecta*, tormentil extract, haemostasis, thrombosis, platelet, bradykinin

## Abstract

*Potentilla* species that have been investigated so far display pharmacological activity mainly due to the presence of polyphenols. Recently, it was shown that polyphenol-rich extract from rhizome of *Potentilla erecta* (tormentil extract) affects the metabolism of arachidonic acid and exerts both anti-inflammatory and anti-oxidant activities, suggesting a possible effect on thrombosis. Accordingly, the aim of the study was to evaluate the effect of tormentil extract on haemostasis in a rat model of thrombosis. Lyophilized water-methanol extract from *P. erecta* rhizome was administrated *per os* for 14 days in doses of 100, 200, and 400 mg/kg in a volume of 2 mL/kg in a 5% water solution of gummi arabici (VEH). In the *in vivo* experiment an electrically induced carotid artery thrombosis model with blood flow monitoring was used in Wistar rats. Collected blood samples were analyzed *ex vivo* functionally and biochemically for changes in haemostasis. Tormentil extract (400 mg/kg) significantly decreased thrombus weight and prolonged the time to carotid artery occlusion and bleeding time without changes in the blood pressure. In the *ex vivo* experiment tormentil extract (400 mg/kg) reduced thromboxane production and decreased t-PA activity, while total t-PA concentration, as well as total PAI-1 concentration and PAI-1 activity remained unchanged. Furthermore, tormentil extract (400 mg/kg) decreased bradykinin concentration and shortened the time to reach maximal optical density during fibrin generation. Prothrombin time, activated partial thromboplastin time, QUICK index, fibrinogen level, and collagen-induced aggregation remained unchanged. To investigate the involvement of platelets in the antithrombotic effect of tormentil, the extract was administrated *per os* for 2 days to mice and irreversible platelets activation after ferric chloride induced thrombosis was evaluated under intravital conditions using confocal microscopy system. In this model tormentil extract (400 mg/kg) significantly reduced platelet activation at the same extent as acetylsalicylic acid. Taken together, we have shown for the first time that tormentil extract inhibits arterial thrombosis in platelet- and endothelial-dependent mechanisms without hemodynamic changes. Further studies on the detailed mechanism of action of tormentil extract toward fibrinolysis and the kinin system should be carried out.

## Introduction

Plants are more and more often taken into consideration as promising sources of new drug candidates for the prevention and treatment of blood-clotting disorders. Numerous studies have demonstrated the ability of active compounds found in plants to reduce platelet activation, increase fibrinolysis or improve endothelial function (Engler et al., 2004; Yoo et al., 2014; Lee et al., 2015; Mohd Nor et al., 2016). Of much importance is the fact that polyphenols possess antithrombotic properties related to antiplatelet and pro-fibrinolytic action (Vita, 2005; Quiñones et al., 2013). A number of epidemiological studies have demonstrated that consumption of polyphenolic compounds limits the incidence of cardiovascular diseases (Yamagata et al., 2015).

The genus *Potentilla* (Rosaceae family), commonly referred to as cinquefoils, consists of about 700 species of annual, and less commonly biennial, plants, and small shrubs (Tomczyk and Latté, 2009). *Potentilla* species which have been investigated so far display a wide spectrum of pharmacological activity mainly due to the presence of polyphenols and triterpenes (Tomczyk and Latté, 2009). However, the influence of the extracts obtained from *Potentilla* plants on haemostatic parameters has not been investigated so far. Polyphenol-rich extract from underground parts of *Potentilla erecta* (tormentil extract) has been used in traditional medicine for the symptomatic treatment of unspecified diarrhea and mild inflammation of the oral and pharyngeal mucosa (Tomczyk and Latté, 2009). This effect is largely associated with its anti-inflammatory and antioxidant properties. On the grounds of the extensively discussed role of inflammation (Aksu et al., 2012) and oxidative stress (Ogita and Liao, 2004) in haemostasis and thrombus development, an evaluation of the antithrombotic properties of tormentil extract seems to be reasonable. Moreover, our preliminary studies (Marcinczyk et al., 2016a,b) showed undefined antithrombotic potential of polyphenolic extract of *P. erecta* rhizome in an electrically induced arterial thrombosis model in rat. Furthermore, Tunón et al. (1995) indicated that tormentil extract inhibits metabolism of arachidonic acid (AA), whose metabolites affect thrombus formation mainly by platelets activation or inhibition (Harlan and Harker, 1981). Wherefore, in our study the influence of tormentil extract on platelet activity has been established *ex vivo* and under intravital conditions. Moreover, the action of tormentil extract toward coagulation cascade and fibrinolysis parameters has been evaluated.

## Materials and Methods

### Preparation of Tormentil Extract

The plant material [*Tormentillae rhizoma*, Ph. Eur. 8.0; *P. erecta* (L.) Raeusch, Rosaceae] was purchased from KAWON (Poland). Accurately weighed 2.0 g of powdered plant material was separately extracted with 150 mL of 80% (*v*/*v*) methanol in an ultrasonic bath (Sonic-5, POLSONIC, Poland) at a controlled temperature (40 ± 2°C) for 45 min. After solvent evaporation under reduced pressure and vacuum controlled temperature (Büchi System, Switzerland) (temperature: 40 ± 2°C) the extract was suspended in water and subjected to lyophilization using a LABCONCO vacuum concentrator (LABCONO, United States) until a constant weight of the extract (yield: 28.97%) was obtained. Dried tormentil extract were kept in an airtight container at 4°C.

### LC–MS Analysis

The LC-ESI-MS analysis of the obtained extract was performed using a 1260 Infinity LC system (Agilent Technologies, United States) equipped with a variable wavelength detector and a 6230 TOF LC/MS mass spectrometer with a Dual Agilent Jet Stream ESI interface (Agilent Technologies, United States). LC analyses of samples were carried out on a reversed-phase Kinetex XB-C18, 150 mm × 3 mm × 2.6 μm column (Phenomenex, United States). The oven temperature was kept at 25°C. The mobile phase (A) was water/formic acid (100:0.1, *v*/*v*) and the mobile phase (B) was acetonitrile/formic acid (100:0.1, *v*/*v*). A two-step gradient system was used: 0–70 min 5–26% B, 70–80 min 26–65% B. The flow rate was 0.400 mL/min. The column was equilibrated for 10 min between injections. Chromatograms were acquired at 254 nm. Compounds were analyzed in negative ion mode. The nebulizer pressure was 45 psi; dry gas flow 12 L/min; dry temperature 300°C; and capillary voltage 2.5 kV. Analysis was carried out using scan from m/z 150 to 2.200.

### Animals

Ninety normotensive male Wistar rats (250–300 g) and 22 male wild type C57BL6 mice (20–23 g) were used in the experiment. Animals were housed in group cages as appropriate, in a room with a 12 h light/dark cycle, and allowed to have access to tap water and a standard rat/mice food. Animals were assigned to control and treated groups randomly. Procedures involving animals and their care were conducted in conformity with the institutional guidelines that are in compliance with national and international laws and Guidelines for the Use of Animals in Biomedical Research. Before arterial thrombosis induction and blood pressure measurement rats were anesthetized with pentobarbital (Morbital, Biovet, Poland, 50 mg/kg, *i.p.*). Wild type mice were anesthetized with ketamine and xylazine (120 mg/kg, *i.p.*, Ketamina 10%, Biowet, Poland; 12.5 mg/kg, *i.p.*, Xylapan, Biowet, Poland) before thrombosis induction. After the experiments animals were killed by cervical dislocation. All the procedures involving the animals and their care were approved by the Local Ethical Committee on Animal Testing at the Medical University of Bialystok (Permit Number: 114/2015, 2/2016). The number of animals used in each experiment is indicated in figure/table description. The 3R rule (“Replacement Reduction and Refinement”) was respected in the study.

### Tormentil Extract Administration

Tormentil extract was administrated *per os* with an oral gastric tube, twice daily for 14 days to rats or for 2 days to mice in doses of 100, 200, or 400 mg/kg in a volume of 2 mL/kg in 5% water solution of gummi arabici (VEH). The last dose of tormentil extract was administrated 30 min before experiments. According to the 3R rule, experiments were performed with a range of tormentil extract doses, while the additional experiments were done only with the effective, highest dose of 400 mg/kg.

### Arterial Thrombosis

Rats were anesthetized, then placed in a supine position on a heated (37°C) surgical table. Arterial thrombosis was induced by electrical stimulation of the right common carotid artery as previously described (Wojewodzka-Zelezniakowicz et al., 2006). Briefly, the anode, a stainless steel L-shaped wire, was inserted under the artery and connected with a constant current generator. The cathode was attached subcutaneously to the hind limb. The artery stimulation (1 mA) took 10 min, then thrombus progression led to a gradual reduction of carotid blood flow (CBF). CBF was monitored in randomly chosen animals only from VEH and 400 mg/kg treated group (3R rule) with a Doppler flow probe (HugoSachs Elektronik, Germany), placed in contact with the exposed artery, downstream of the electrode, and connected to a blood flowmeter (HugoSachs Elektronik, Germany). Blood flow was monitored continuously during the entire study. The occlusion was defined as lack of arterial blood flow. The total time to occlusion was defined as the time from the start of electrical stimulation until the occlusion. 55 min after the commencement of the stimulation, the segment of the common carotid artery with the formed thrombus was dissected, then opened lengthwise and the thrombus was completely removed, dried at room temperature and weighed after 24 h. For *ex vivo* experiments, blood samples were collected from the left heart ventricle after thrombus removal using 3.13% sodium citrate solution (1:10, *v*/*v*) as an anticoagulant.

### Blood Pressure Measurements

Rats were anesthetized and placed in a supine position on a heated (37°C) surgical table. The systolic and diastolic blood pressure were measured directly through a cannula filled with heparin solution (150 IU/ml), placed in the left common carotid artery and connected to a pressure transducer (Gabarith 1DT-XX, Poland), and an apparatus for pressure measurement (HSE TRANSONIC, TAM-A, Germany), as described previously (Wojewodzka-Zelezniakowicz et al., 2016).

### Platelet Aggregation

Collagen-stimulated (5 μl/ml) platelet aggregation (Chrono-Par Collagen, United States) in 500 μl of citrated whole blood was evaluated *ex vivo* with the impedance method as described previously (Kramkowski et al., 2016) and measured in a Whole Blood Lumi-Aggregometer (Chrono-log, Corp., United States).

### Dynamic Generation of Thromboxane B_2_ (TXB_2_)

Dynamic generation of thromboxane was performed as described previously (Kramkowski et al., 2016). Briefly, TXB_2_, a stable metabolite of TXA_2_, was generated in 500 μl of citrated whole blood samples diluted with 0.9% NaCl in a 1:1 ratio and vigorously stirred in aggregation cuvettes at 37°C at 1000 rpm. After 60 min 200 μl of stirred blood were drawn off and mixed with 200 μl of cold solution of acetylsalicylic acid (ASA) (final concentration 500 mM) to stop further TXB_2_ generation. The concentration of TXB_2_ was analyzed in the obtained samples using an ELISA kit (Enzo Life Sciences, United Kingdom) and a microplate reader ELx808 (BioTek Instruments, Inc., United States).

### Bleeding Time

Bleeding time was performed as described previously prior to the arterial thrombosis induction. Briefly, a standardized device was applied longitudinally on the dorsal part of the tail between 6 and 9 cm from the tip, taking care to avoid the large veins. Immediately after injury, the tail was placed into a cylinder with isotonic saline at 37°C and bleeding time was measured from the moment the tail was surgically cut until bleeding completely stopped (lack of bleeding for < 30 s) (Wojewodzka-Zelezniakowicz et al., 2016).

### Intravital Observation of Platelets Activity in a Mouse Mesenteric Vein after Acute Administration of Tormentil Extract

We used fixed-stage microscope Zeiss Axio Examiner Z.1 (Carl Zeiss Microscopy GmbH, Germany) to intravital observation of the platelets activity within the ferric chloride (FeCl_3_) induced thrombus in a wild type mouse mesenteric vein. ASA in a dose of 100 mg/kg (Flectadol 1 g/5 ml, Sanofi, Italy) as a positive control was injected into the left femoral vein 5 min before endothelial injury. To visualize irreversible platelet activation Alexa Fluor 647-labeled annexin V (ANX; 2 μg/g of mouse body weight in 0.1 ml PBS, Alexa Fluor^TM^ 647 annexin V conjugate, Thermo Fisher Scientific, United States) was injected into the right femoral vein 5 min before the endothelial injury. To visualize vessel wall, partially activated platelets and non-activated platelets the DiOC6(3) [0.1 mM in 0.05 ml of the mixture of DMSO and PBS (volume ratio 1:50), Life Technologies, Molecular Probes, United States] was administrated with intramuscular injection 5 min before endothelial injury. A midline laparotomy incision was made, and then the mesentery of the ileum was pulled out of the abdomen and draped over a plastic mound. 0.1 μL of 20% solution of FeCl_3_ was administrated topically at the mesenteric vein and immediately wash out with PBS. The mesentery vein was placed under the objective (W Plan-Apochromat 20×/1.0 water immersion objective, Carl Zeiss Microscopy GmbH, Germany) and identified. The mesentery was continuously perfused with 37°C-warmed PBS to prevent the vessels from drying. During the experiment Alexa Fluor^TM^ 647 was excited by 640 nm laser and DiOC6(3) was excited by 488 nm laser (LaserStack 488 nm, LaserStack 640 nm, 3iL33, Intelligent Imaging Innovations, Inc., United States). The thrombus development was registered for 6 min with Confocal Scanner Unit CSU-X1 (Yokogawa Electric Corporation, Japan) in one focal plane (2D imaging) corresponding with the largest area of the thrombus. At most two thrombi were induced in one mouse. Collected images (four images per second) were analyzed using SlideBook 6 (Intelligent Imaging Innovations, Inc., United States). The regions of red fluorescence (regions correspond with irreversible activated platelet) were encircled every 30 s of record in particular image. The areas of regions from one thrombus were calculated with SlideBook 6 and then added up.

### Fibrinolysis Parameters

Total plasminogen activator inhibitor-1 antigen (PAI-1), active PAI-1, total tissue plasminogen activator antigen (t-PA) and active t-PA were analyzed using ELISA kits (Innovative Research, Inc., United States) and a microplate reader ELx808 (BioTek Instruments, Inc., United States).

### Fibrin Generation

Fibrin generation was initiated by recalcination of plasma samples directly in microplate wells with CaCl_2_ (36 mmol/L) dissolved in Tris buffer (66 mmol/L Tris; 130 mmol/L NaCl; pH = 7.4) at 37°C. Increasing optical density in the wells (as a result of fibrin generation) was measured *via* the ELx808 microplate reader (BioTek Instruments, Inc., United States) in 1-min intervals for 20 min. The rate of fibrin generation was expressed as the time (min) to reach maximal optical density in each well.

### Bradykinin Concentration

Bradykinin (BK) concentration was analyzed using ELISA kits (Enzo Life Sciences, United Kingdom) and the ELx808 microplate reader (BioTek Instruments, Inc., United States).

### Coagulation Parameters and Blood Cell Count

The activated partial thromboplastin time (APTT), prothrombin time (PT), also expressed as the QUICK index, and INR and fibrinogen concentration were determined automatically using the Coag-Chrom 3003 apparatus (Bio-ksel, Poland) and standard dedicated laboratory reagents (Bio-ksel, Poland). Blood count was performed using a ScilVet ABC Plus+ hematological analyzer (HORIBA ABX, France). The counting of blood cells was based on the volumetric impedance method.

### Statistical Analysis

The data are shown as mean ± SEM or median (interquartile range) and analyzed using either the Student’s *t*-test (when the normality test passed) or the Mann–Whitney test (when the normality test failed). *p* < 0.05 was considered significant. All analyses were done in GraphPad Prism 5.

## Results

### LC–MS Analysis

We initiated a detailed phytochemical analysis of the secondary metabolites present in the investigated tormentil extract, and confirmed the presence of polyphenolic compounds. The fingerprints of the analyzed tormentil extract were established using the LC-ESI-MS method. The analysis revealed the presence of 17 constituents comprising proanthocyanidins, flavan-3-ol oligomers (3–10, 12, 13), as well as hydrolysable ellagitannins and also related compounds (1, 2, 11, 14–17). The dominating compounds of the tested extract are oligomeric proanthocyanidins (3, 4) and agrimoniin (16). The main tormentil proanthocyanidins are B-type procyanidins with catechin and epicatechin units. The minor phytoconstituents of the analyzed extract were glycoside derivatives of ellagic acid. Fecka et al. (2015) reported that the main components of the tormentil tinctures were oligomeric tannins with an average content of 5.26 mg/g, procyanidin C2 (3.36 mg/g) and agrimoniin (2.05 mg/g). **Table 1** contains detailed UV-VIS and MS data for all the detected compounds together with their preliminary or full identification.

**Table 1:** MS and UV-VIS data of compounds with their retention times detected in the extract prepared from the rhizomes of *Potentilla erecta.*

Analytes	R_t_ [min]	UV_max_ [nm]	[M-H] *m/z*
1. Pedunculagin α or β	5.9	241	783
2. Pedunculagin α or β	10.3	241	783
3. Procyanidin B3	11.9	242, 278	577/1155
4. Procyanidin C2	13.6	242, 278	865/1732
5. PC tetramer	18.8	242, 278	1153
6. PC trimer	19.7	242, 278	865
7. PC trimer	20.9	242, 278	865
8. PC dimer	21.2	242, 278	577/1155
9. PC tetramer	24.2	242, 278	1153
10. PC pentamer	25.5	242, 279	1141/1153
11. Laevigatin (dimer)	28.6	245	1567/783
12. PC tetramer	30.8	242, 278	1153
13. PC trimer	32.9	244, 277	865
14. Methylellagic acid glucuronide	33.9	244, 275	983/491
15. Laevigatin (dimer)	34.2	244, 276	1567/783
16. Agrimoniin	41.7	264	1870/934
17. Methylellagic acid pentoside	44.9	248, 364	447/895

*M-H, molecular ion of the formula in negative ionization mode*.

### General Characteristics of Animals

The survival rate of rats was not affected by tormentil extract treatment (VEH: 97%, 100 mg/kg: 100%, 200 mg/kg: 100%, 400 mg/kg: 93%, *p* = ns). Increase in body weight after 14 days of tormentil extract treatment was similar in all groups (VEH: 1st day: 216 ± 4, 14th day: 274 ± 5, 100 mg/kg: 1st day: 202 ± 5, 14th day: 253 ± 6, 200 mg/kg: 1st day: 206 ± 4, 14th day: 260 ± 7, 400 mg/kg: 1st day: 216 ± 5, 14th day: 264 ± 6, *n* = 20–22, *p* = ns).

### Arterial Thrombosis

**FIGURE 1:**
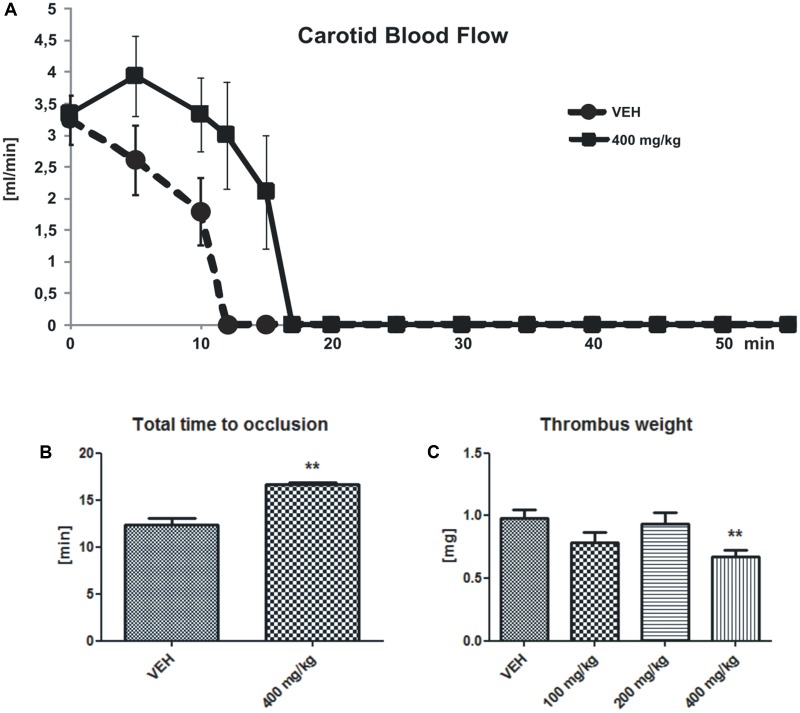
Changes in the carotid blood flow (CBF) **(A)** determined in rats subjected to electrical stimulation. The lines represent the course of blood flow registered for 55 min; *n*: VEH = 4, 400 mg/kg = 5, *p* = ns. The effect of tormentil extract treatment on the total time to occlusion **(B)** (^∗∗^*p* < 0.01 vs. VEH, *n*: VEH = 4, 400 mg/kg = 5) and dry thrombus weight **(C)** ^∗∗^*p* < 0.01 vs. VEH, *n*: VEH = 18, 100 mg/kg = 11, 200 mg/kg = 16, 400 mg/kg = 19. Data are shown as mean ± SEM.

There were no changes of the initial CBF between the group with the highest dose of tormentil extract (400 mg/kg) and the control group (3.34 ± 0.89 mL/min vs. VEH 3.23 ± 0.39 mL/min, **Figure 1A**), while tormentil extract in a dose of 400 mg/kg significantly prolonged the total time to occlusion (16.67 ± 0.19 min vs. VEH 12.05 ± 0.71 min, *p* < 0.01, **Figure 1B**) and decreased thrombus weight (0.68 ± 0.05 mg vs. VEH 0.98 ± 0.07 mg, *p* < 0.01, **Figure 1C**).

### Bleeding Time

**FIGURE 2:**
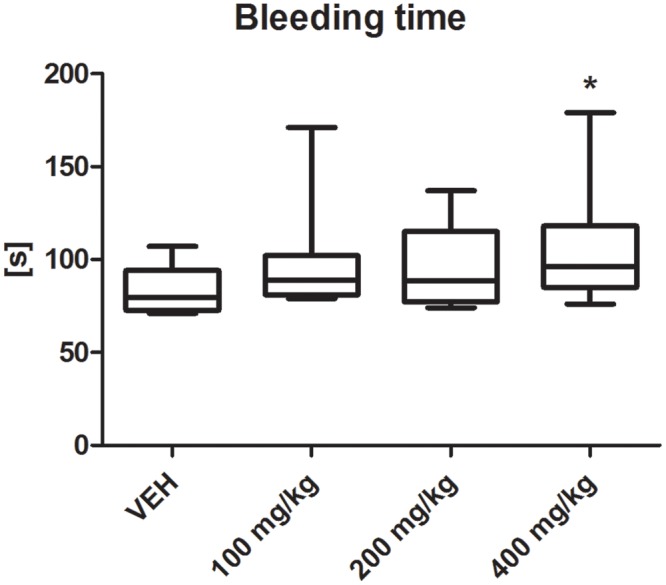
Effect of tormentil extract on bleeding time; ^∗^*p* < 0.05 vs. VEH, *n*: VEH = 9, 100 mg/kg = 10, 200 mg/kg = 11, 400 mg/kg = 12. Data are shown as median (interquartile range).

Tormentil extract in a dose of 400 mg/kg prolonged bleeding time [96 (85;118) s vs. VEH 79.5 (73;94) s, *p* < 0.05, **Figure 2**].

### Blood Pressure

**FIGURE 3:**
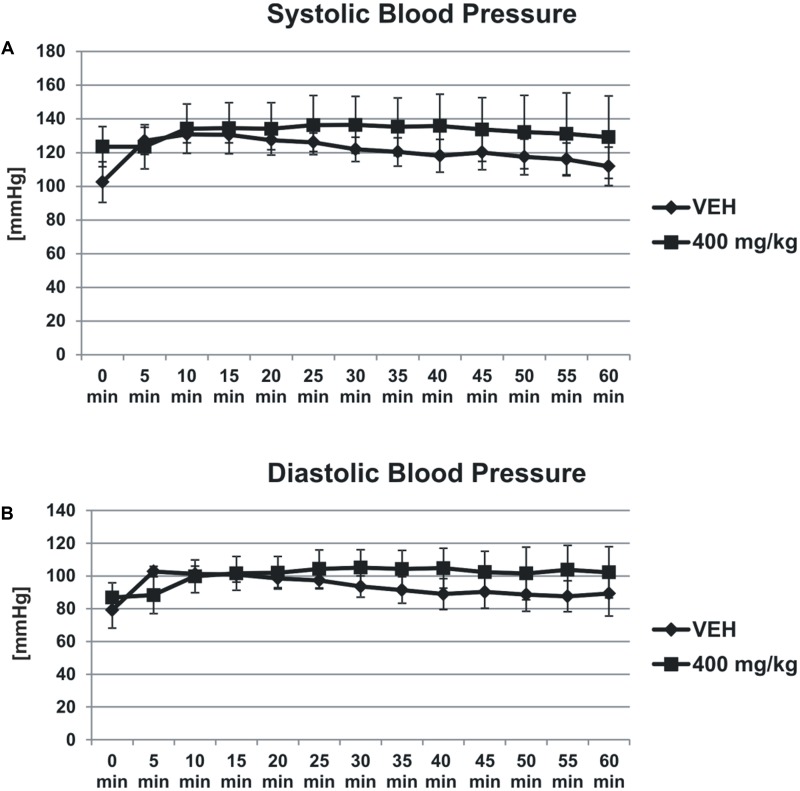
Effect of tormentil extract on systolic **(A)** and diastolic **(B)** blood pressure. The lines represent the changes in the blood pressure registered for 60 min; *p* = ns, *n* = 3. Data are shown as mean ± SEM.

Tormentil extract in a dose of 400 mg/kg had no effect on systolic (1st min: 124 ± 11 mmHg, 60th min: 129 ± 24 mmHg vs. VEH 1st min: 102,5 ± 12 mmHg, 60th min: 112 ± 11, **Figure 3A**) and diastolic (1st min: 87 ± 9 mmHg, 60th min: 102 ± 15 mmHg vs. VEH 1st min: 79 ± 11 mmHg, 60th min: 89 ± 14 mmHg, **Figure 3B**) blood pressure.

### Platelet Function

**FIGURE 4:**
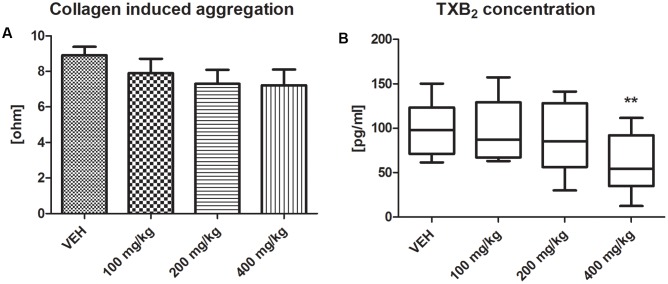
Effect of tormentil extract on collagen-induced aggregation **(A)**. Bars represent values of impedance; *n*: VEH = 11, 100 mg/kg = 9, 200 mg/kg = 9, 400 mg/kg = 11, *p* = ns. Data are shown as mean ± SEM. Thromboxane B_2_ concentration after 60 min of experiment **(B)**; ^∗∗^*p* < 0.01 vs. VEH, *n*: VEH = 11, 100 mg/kg = 9, 200 mg/kg = 10, 400 mg/kg = 12. Data are shown as median (interquartile range).

Tormentil extract in a dose of 400 mg/kg had no effect on collagen-induced aggregation in whole blood (7.2 ± 0.4 ohm vs. VEH 8.9 ± 0.5 ohm, *p* = 0.09, **Figure 4A**), but significantly reduced platelet TXB_2_ generation [54.30 (34.90;92.15) pg/mL vs. VEH 97.75 (71.08;123.40) pg/mL, *p* < 0.01, **Figure 4B**].

### Intravital Observation of Platelet Activity in a Mouse Mesenteric Vein after Acute Administration of Tormentil Extract

**FIGURE 5:**
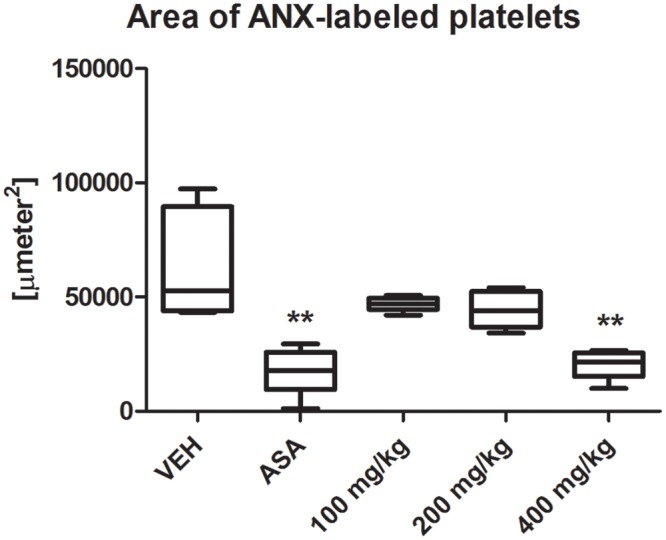
Effect of tormentil extract and ASA on irreversible platelet activation in ferric chloride induced thrombosis in mouse. The bars represent area of ANX-labeled irreversible activated platelets within ferric chloride induced thrombus. ^∗∗^*p* < 0.01 vs. VEH, *n*: VEH = 7, ASA = 7, 100 mg/kg = 7, 200 mg/kg = 7, 400 mg/kg = 12. Data are shown as median (interquartile range).

Tormentil extract in a dose of 400 mg/kg reduced platelet activity at the same extent as ASA in a dose of 100 mg/kg [21591 (15195;25531) μm^2^, ASA: 17721 (9382;25778) μm^2^ vs. VEH 52765 (43900;89544) μm^2^, *p* < 0.01, **Figure 5**]. Picture in the **Supplementary Figure S1**.

### Fibrinolysis Parameters

**FIGURE 6:**
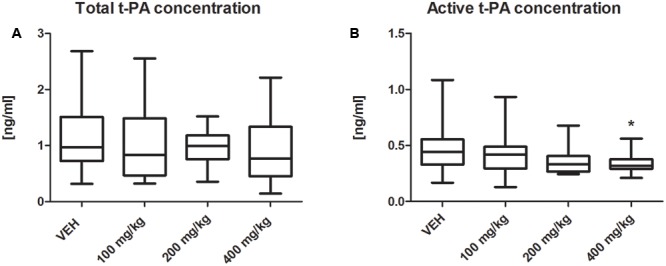
Effect of tormentil extract on total **(A)** and active **(B)** concentration of t-PA; ^∗^*p* < 0.05 vs. VEH, *n*: VEH = 11, 100 mg/kg = 9, 200 mg/kg = 10, 400 mg/kg = 12. Data are shown as median (interquartile range).

The t-PA activity was significantly decreased in the plasma of rats treated with tormentil extract in a dose of 400 mg/kg compared to non-treated rats [0.32 (0.29;0.56) ng/mL vs. VEH 0.48 (0.43;0.55) ng/mL, *p* < 0.05, **Figure 6B**], while total t-PA concentration was unchanged [0.76 (0.45;1.33) ng/mL vs. VEH 0.97 (0.73;1.50) ng/mL, *p* = 0.22, **Figure 6A**]. Furthermore, both total PAI-1 concentration and PAI-1 activity were not affected by tormentil extract (**Table 2**).

**Table 2:** Values of coagulation parameters, fibrinolysis parameters, and blood cell count.

Coagulation parameters				
**Group**	**VEH *n* = 20**	**100 mg/kg *n* = 15**	**200 mg/kg *n* = 18**	**400 mg/kg *n* = 21**

PT [s]	12.04 ± 0.60	12.31 ± 0.40	12.88 ± 0.20	12.02 ± 0.40
QUICK [%]	94.7 ± 3.3	90.3 ± 2.5	86.3 ± 1.2	93.6 ± 3
INR	1.10 ± 0.10	1.10 ± 0.03	1.16 ± 0.02	1.08 ± 0.03
Fibrinogen [g/l]	2.02 ± 0.05	2.25 ± 0.08	2.24 ± 0.20	2.14 ± 0.08
APTT [s]	16.54 ± 0.60	17.56 ± 0.99	16.83 ± 0.48	17.14 ± 0.22

**Fibrinolysis parameters**				

**Group**	**VEH *n* = 11**	**100 mg/kg *n* = 9**	**200 mg/kg *n* = 10**	**400 mg/kg *n* = 12**

PAI-1 total [ng/ml]	1.16 ± 0.10	1.28 ± 0.15	1.10 ± 0.06	1.30 ± 0.11
PAI-1 activity [ng/ml]	1.50 ± 0.22	1.25 ± 0.30	1.29 ± 0.14	1.67 ± 0.23

**Blood cell count**				

**Group**	**VEH *n* = 20**	**100 mg/kg *n* = 13**	**200 mg/kg *n* = 21**	**400 mg/kg *n* = 22**

WBC [10^3^ mm^3^]	2.8 ± 0.2	3.1 ± 0.4	2.3 ± 0.2	2.9 ± 0.2
RBC [10^6^ mm^3^]	7.3 ± 0.2	7.3 ± 0.1	7.2 ± 0.9	7.4 ± 0.1
HGB [g/dl]	14.4 ± 0.2	14.3 ± 0.2	14.0 ± 0.3	14.4 ± 0.2
HCT [%]	42.0 ± 0.6	42.0 ± 0.6	41.3 ± 0.8	42.0 ± 0.7
PLT [10^3^ mm^3^]	557.2 ± 20	572.0 ± 16.9	590.2 ± 14	588.0 ± 17.1
MCV [fl]	57.3 ± 0.3	57.5 ± 0.4	57.5 ± 0.5	56.9 ± 0.4
MCH [pg/cell]	19.9 ± 0.2	19.5 ± 0.1	19.5 ± 0.2	19.5 ± 0.2
MCHC [g/Dl]	34.7 ± 0.3	34.0 ± 0.1	33.8 ± 0.2	34.3 ± 0.3

*p = ns. Data are shown as mean ± SEM*.

### Bradykinin Concentration and Fibrin Generation

**FIGURE 7:**
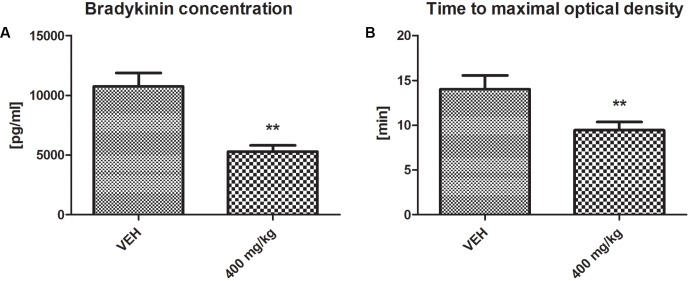
Effect of tormentil extract on bradykinin concentration **(A)**; ^∗∗^*p* < 0.01 vs. VEH, *n* = 7. Data are shown as mean ± SEM. Fibrin generation after plasma recalcination **(B)**; bars represent the time to reach maximal optical density; ^∗∗^*p* < 0.01 vs. VEH, *n* = 7. Data are shown as mean ± SEM.

The bradykinin concentration was significantly decreased in the plasma of rats treated with tormentil extract in a dose of 400 mg/kg compared to non-treated rats (5299 ± 507 pg/ml vs. VEH 10743 ± 1120 pg/ml, *p* < 0.01, **Figure 7A**). Tormentil extract in a dose of 400 mg/kg shortened the time to reach maximal optical density due to fibrin formation (9.44 ± 0.91 min vs. VEH 14.01 ± 1.54 min, *p* < 0.01, **Figure 7B**).

### Coagulation Parameters and Blood Cell Count

Tormentil extract failed to affect coagulation parameters. Values of PT, QUICK, INR, fibrinogen level and APTT are shown in **Table 2**. No changes were observed in blood cell count in animals treated with tormentil extract, or in non-treated animals (**Table 2**).

## Discussion

In our study we have shown for the first time the *in vivo* antithrombotic effect of tormentil extract in the rat model of electrically induced arterial thrombosis after 14 days of treatment. In our study tormentil extract prolonged the time to occlusion in the thrombotic vessel, decreased thrombus weight and prolonged bleeding time without hemodynamic changes. Tormentil extract reduced *ex vivo* thromboxane generation by platelets. In intravital experiment employing confocal microscopy system tormentil extract reduced platelets activity within ferric chloride induced thrombus in a mouse mesentery vein after 2 days of administration. Furthermore, fibrin generation after plasma recalcination was enhanced and BK concentration and active t-PA concentration were decreased. Coagulation parameters: APTT, PT, QUICK, INR, fibrinogen level and the blood cell count remained unchanged. The small drop in the survival rate (3–7%) was the result of perioperative complications (incidental injury of blood vessel with needle or surgical equipment), though rats gained approximately 50 g after 14 days of treatment. Taken together, all these findings preliminarily indicate the high therapeutic potential of tormentil extract without toxic effects.

In the present work we used an electrical-induced model of arterial thrombosis. This model evaluates blood flow disturbances as a result of growing, vessel occluding thrombus after extensive endothelium damage. This imitates clinically relevant situations like myocardial infarction. Indeed, tormentil extract at the highest dose prolonged the time to carotid artery occlusion and significantly reduced thrombus weight. In the same experiment the initial blood flow was not changed after tormentil extract treatment and the gradual decrease in the blood flow due to thrombus formation, as well as occlusion incidences, were observed both in the control and the tormentil extract treated group. Since the values of initial CBF and blood pressure were not significantly changed after treatment we presumed that tormentil extract did not affect the blood vessel tone.

In turn, our earlier studies (Kramkowski et al., 2006; Wojewodzka-Zelezniakowicz et al., 2006, 2016) clearly showed that angiotensin converting enzyme inhibitors (ACE-Is) improve endothelial function in a mechanism involving, among others, BK increase. In the same model of thrombosis, ACE-Is decreased thrombus weight, prolonged the time to carotid artery occlusion, increased blood flow distally to the place of vessel injury and decreased collagen-stimulated whole blood platelet aggregation. To elucidate the antithrombotic effect of tormentil extract in spite of BK decrease, we performed more detailed experiments.

In the model of electrically induced thrombosis, the mechanism of thrombus formation is mainly related to the platelets accumulating at the subendothelial matrix exposed due to the electrical injury. Furthermore the bleeding time method is used to investigate the primary haemostasis. After vessel wall injury and following vasoconstriction platelets accumulate at the site of the injury to form platelet plug (Wojewodzka-Zelezniakowicz et al., 2016). Prolonged bleeding time clearly indicate the involvement of platelets inhibition in the antithrombotic effect of tormentil extract. To determine the influence of tormentil extract on platelets activation, first we used a method of collagen-induced platelet aggregation in whole blood. Again, contrary to ACE-Is, tormentil extract showed a slight but not statistically important anti-aggregatory effect. Since BK in the calmodulin-dependent mechanism increases endothelial NOS expression, the strong antiplatelet effect of ACE-Is in rat seems to be clear. On the other hand, in human platelet collagen causes robust platelet activation and leads to the release of agonists (e.g., TXA_2_ or ADP) which potentiate the aggregation (Nieswandt and Watson, 2003), while rat platelets are less reactive to collagen and require higher doses to induce aggregation (Emms and Lewis, 1986). Thus, the method of collagen-induced aggregation is adequate to demonstrate considerable changes in platelet responses (Takahashi, 2000) like NO dependent effects. The metabolism of derivatives of methylellagic acid, present in the tormentil extract, leads to the production of urolithins which inhibit inducible NOS (Espín et al., 2013). Literature data show that BK upregulate the pro-inflammatory iNOS gene (Savard et al., 2008). It is more likely that the antiplatelet effect of tormentil extract is not NO-dependent. Furthermore, collagen (a potent platelet agonist) triggered many signaling pathways *via* multiple receptors simultaneously, and masked subtle differences in platelets’ responses in our experiment.

To elicit weak platelet activation we used an assay of dynamic thromboxane B_2_ (TXB_2_) generation where its concentration was assessed in whole blood after 60 min of vigorous stirring (Kramkowski et al., 2016). Indeed, thromboxane A_2_ (TXA_2_) is a potent platelet agonist *in vivo* produced *via* AA cascade in a response to variable stimuli, e.g., collagen (Nieswandt and Watson, 2003), shear stress (Kramkowski et al., 2016). In this assay TXB_2_ (stable metabolite of TXA_2_), liberated from platelets, was recognized as a marker of their activity. Regarding the previously described inhibitory effect of tormentil extract on AA metabolism (Tunón et al., 1995; Hoffmann et al., 2016) we chose one of its metabolites (TXA_2_) to demonstrate tormentil extract action toward AA cascade in platelets. Platelet- derived COX-1 is responsible for the generation of TXA_2_, while COX-2 pathway leads to the production of pro-inflammatory prostaglandins, including prostaglandin E_2_ (PGE_2_) (Harlan and Harker, 1981). The inhibitory effect of tormentil extract (especially its agrimoniin enriched fraction) on COX-2 expression and the production of PGE_2_ in human keratinocytes was reported (Hoffmann et al., 2016), but the influence of the tormentil extract on COX-1 expression and TXA_2_ production has not yet been investigated. We found that TXB_2_ concentration in blood obtained from rats treated with the highest dose of tormentil extract was significantly lower compared to the control group. This indicates that the antiplatelet activity of tormentil extract is at least partially related to the inhibition of thromboxane production. It is most likely that the inhibition of thromboxane synthesis by tormentil extract contributed to the attenuation of collagen-induced aggregation, but this effect was not sufficient to achieve a significant decrease.

To investigate the influence of tormentil extract on the irreversible platelets activation we used confocal microscopy system to intravital observation of the platelets activity within ferric chloride induced thrombus. In this method platelets accumulate to the collagen exposed after endothelium damage (Rybaltowski et al., 2011). Contact with collagen leads to the increase of intracellular calcium concentration and reorganization of the platelet cytoskeleton. This induces changes in a platelet shape followed by exposure of the phosphatidylserine from the inner to the outer leaflet. Phosphatidylserine is present on the surface of irreversible activated platelets at the core of thrombus and provides catalytic surface for procoagulant reactions. To visualize irreversible activated platelets we used Alexa Fluor 647-labeled annexin V which is protein with strict specificity to phosphatidylserine (Hayashi et al., 2008). DiOC6(3) was injected to visualize vessel wall and platelets without phosphatidylserine exposed on their surface. To investigate the influence on platelets, tormentil extract was administrated only for 2 days to avoid its effect on endothelial enzymes. Tormentil extract in a dose of 400 mg/kg decreased the area of irreversible activated platelets within the ferric chloride induced thrombus at the same extent as ASA which is a platelet COX-1 inhibitor (Harlan and Harker, 1981).

Taken together with the slight anti-aggregatory effect, this potent antiplatelet action suggests the important involvement of platelets and the modification of AA metabolism in the mechanism of tormentil extract action.

Furthermore, we assessed the influence of tormentil extract on fibrinolysis, showing decreased concentration of active t-PA in rats treated with tormentil extract in a dose of 400 mg/kg. In the face of attenuated thrombosis and unchanged values of total PAI-1 concentration and PAI-1 activity, the reduction of t-PA activity, which is responsible for the conversion of plasminogen to plasmin, was surprising. Supportive literature data suggest that the reduction of active t-PA concentration may be due to the interference of tormentil extract with the kinin system. It has been shown that intravenous administration of BK resulted in the increased level of t-PA in rat plasma (Smith et al., 1985). Thus, we considered that tormentil extract may interact with the kinin system. Using the ELISA test we estimated that BK level was significantly decreased in plasma obtained from rats treated with the highest dose of tormentil extract. This could explain the reduction of t-PA activity, but the exact mechanism of this action needs to be evaluated in the future. Although the major fibrinolysis parameters have been measured, no clear conclusion can be made about clot dissolution yet. Thus the euglobulin clot lysis time (ECLT) is required to assess the net activity of tormentil extract on the fibrinolysis process. Moreover, in the *in vitro* study using the Western blot technique it has been demonstrated that the incubation of high molecular weight kininogen (HMWK) with t-PA and plasminogen leads to the cleavage of HMWK to BK (Marcos-Contreras et al., 2016). HMWK is a part of the plasma contact activation system (CAS) and enhances the reciprocal activation of prekallikrein and factor XII to their active forms to produce thrombin on the way which was formerly known as an intrinsic pathway (Schmaier, 2014). Since we observed decreased levels of BK, it is possible that tormentil extract modulates the contact activation of coagulation through the inhibition of HMWK cleavage. The APTT is used to evaluate factors of CAS (HMWK, prekallikrein), intrinsic pathway and common pathway (Levy et al., 2014). Nevertheless, in the face of the lack of tormentil extract effect on APTT a more precise experiment on the coagulation cascade was required. For this purpose calcium chloride was added to the citrated plasma to initiate its coagulation. The shortened time to reach maximal absorbance due to fibrin formation in the group treated with tormentil extract in a dose of 400 mg/kg indicates that tormentil extract modulates coagulation cascade and enhances fibrin generation. This effect is probably related to the increased level of HMWK, which augments the pathway of CAS. However, this assay is not specific, and the determination of HMWK with ELISA is required. Furthermore, Kurata et al. (2003) demonstrated that increased concentration of fibrinogen did not affect the APTT in rats. This could explain the unchanged values of APTT despite enhanced fibrin generation. Besides the modulation of fibrinolysis and coagulation cascade, the effect of decreased BK concentration on haemostasis could be due to the suppression of pro-inflammatory responses. Bradykinin promotes the expression of the vascular cell adhesion molecule-1 (VCAM-1) and induces the release of cytokines such as tumor necrosis factor α (TNF-α) and interleukin 1β (IL-1β) (Chu, 2010). The reduction of these effects might contribute to diminished thrombosis in our experiment.

Summarizing, the antithrombotic effect of tormentil extract is complex. Tormentil extract prolonged the time to carotid artery occlusion, decreased thrombus weight and prolonged bleeding time without hemodynamic changes. Tormentil extract reduced thromboxane generation in platelets and decreased area of irreversible activated platelets within thrombus in a mouse mesentery vein. The antiplatelet effect of tormentil extract on ferric chloride induced thrombosis is comparable to antiplatelet effect of ASA in this model. On the other hand, the concentration of the active tissue plasminogen activator was decreased after tormentil extract treatment, but its net effect on fibrinolysis is not yet known. Tormentil extract elevated the fibrin generation rate. However, this action did not abrogate the antithrombotic effect. Tormentil extract decreased the concentration of bradykinin, but the exact mechanism toward the kinin system needs to be evaluated in the future. Taken together, all these findings suggest that *P. erecta* rhizome extract exerts antithrombotic action related to platelet and endothelium-dependent mechanism. However, this endothelial mechanism, contrary to well-known drugs with pleiotropic action, seems to be dependent on bradykinin decrease, not increase. Further investigations are planned, assessing both the mechanism of tormentil extract action, as well as indicating the active component or components of the extract responsible for its antithrombotic potential.

Previous studies have suggested that identified compounds in the tormentil extract such as flavan-3-ol oligomers have a positive influence on the human health. Procyanidin shows obvious anti-thrombotic effect. Its mechanism of action closely correlates with inhibition of platelet activation and aggregation as well as protection of vasoendothelial cells (Jiang et al., 2007). Procyanidin-rich extract obtained from *Vitis vinifera* seeds prolongs bleeding time but does not inhibit platelet adhesion to collagen or thrombosis. It is possible that the prolongation of bleeding time in this test model might be due to the vasodilatory effect of the extract rather than to reduced platelet–vessel wall interactions (De Curtis et al., 2003). Chang and co-authors studied the effects of procyanidins on platelet aggregation and arachidonate metabolism in platelets. Procyanidins significantly induced the inhibition of platelet aggregation, and the potency of the inhibition was comparable with aspirin. The inhibitory effect on thromboxane biosynthesis might explain the inhibitory effect of procyanidin B2 on platelet aggregation (Chang and Hsu, 1989). Also pedunculagin showed significant anticoagulant activity with clotting times greater than heparin. Pedunculagin seemed to be a competitive inhibitors of factor Xa (Dong et al., 1998).

Our study showed that tormentil extract provides a useful preventive approach or an adjunct to current pharmacological treatments for thrombotic diseases.

## Ethics Statement

This study was carried out in accordance with the recommendations of the Local Ethical Committee on Animal Testing at the Medical University of Bialystok and conducted inaccordance with the institutional guidelines, which are in compliance with national and international laws, including EUDirective 2010/63/EU for animal experiments and the Guidelines for the Care and the Use of Animals in Biomedical Research (Giles, 1987).

## Author Contributions

All authors participated in the experiments of the study and review of the manuscript. EC, MT, and KK designed the experiments. MT prepared and supplied tormentil extract. NM, EC, and KK analyzed data and wrote the manuscript. All authors revised and approved the final manuscript.

## Conflict of Interest Statement

The authors declare that the research was conducted in the absence of any commercial or financial relationships that could be construed as a potential conflict of interest.

## Supplementary Material

FIGURE S1 Representative picture from confocal intravital model: fluorescence of vessel wall, partially activated platelets and non-activated platelets labeled with the DiOC6(3) [Green] or irreversible activated platelets labeled with Alexa Fluor 647-labeled annexin V (ANX) [Red] in mice with FeCl_3_-induced thrombosis in control group (‘VEH’), in positive control group (acetylosalicylic acid, 100 mg/kg, ‘ASA’) and in group of the highest dose (400 mg/kg) of tormentil extract (‘400’).
