# Supplementary figures and images for: Antithrombotic Potential of Tormentil Extract in Animal Models
Source: Front Pharmacol. 2017 Aug 15;8:534. doi: 10.3389/fphar.2017.00534 (PMC5559472; doi:10.3389/fphar.2017.00534)

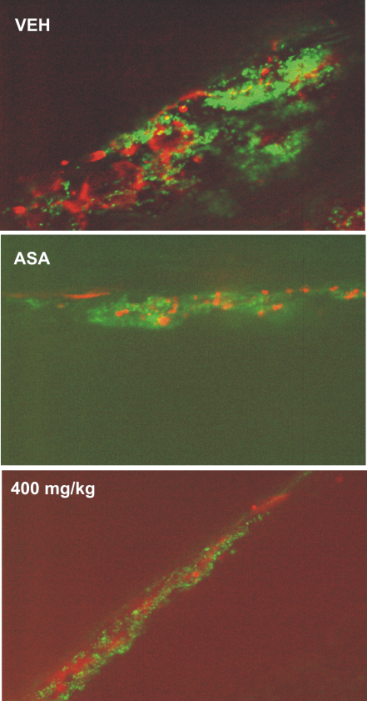

Supplement: FIGURE S1 — Representative picture from confocal intravital model: fluorescence of vessel wall, partially activated platelets and non-activated platelets labeled with the DiOC6(3) [Green] or irreversible activated platelets labeled with Alexa Fluor 647-labeled annexin V (ANX) [Red] in mice with FeCl3-induced thrombosis in control group (‘VEH’), in positive control group (acetylosalicylic acid, 100 mg/kg, ‘ASA’) and in group of the highest dose (400 mg/kg) of tormentil extract (‘400’). [file Image_1.TIF]
